# Educational Outcomes Associated With Undergraduate Dental Education in Primary Care and Community Settings in Europe: Findings From a Rapid Scoping Review

**DOI:** 10.1111/eje.70004

**Published:** 2025-06-30

**Authors:** Paul Leavy, Blánaid Daly

**Affiliations:** ^1^ Centre for Health Policy and Management Trinity College Dublin Dublin 2 Ireland; ^2^ School of Dental Science, Trinity College Dublin Dublin Dental University Hospital Dublin 2 Ireland

**Keywords:** community, dental student, education, primary care, undergraduate

## Abstract

**Background:**

Recent decades have seen the emergence of primary care and community (PCC) based dental education in settings that are separate to dental teaching hospitals, including general dental practice and salaried dental services. By mirroring care delivery in these services, this experience can facilitate students' transition to independent clinical practice.

**Objectives:**

To identify educational outcomes associated with PCC‐based undergraduate dental education in Europe.

**Methods:**

Web of Science, MEDLINE, CINAHL, Academic Search Complete and ERIC were searched, and peer‐reviewed articles were selected based on inclusion criteria. Following data extraction, a narrative synthesis of findings was conducted with outcomes grouped and themed into five domains: ‘practical skills’; ‘teamwork and the business of dentistry’; ‘preparedness for independent practice’; ‘population and dental public health’ and ‘specialty specific outcomes’.

**Results:**

Forty‐seven studies from the UK, Germany, and Nordic countries were included. PCC‐based education was noted for promoting teamwork and affording undergraduates experience in the operation of dental clinics. Improved practical skills relating to treatment planning, provision of holistic care, communication and clinical productivity were observed. Students also gained experience in population and dental public health, paediatric and special care dentistry.

**Conclusions:**

PCC‐based dental education facilitates students in consolidating their learning and honing their skills in preparation for independent practice. The outcomes cited align with many of the objectives and intended learning outcomes of such programmes, as well as recommendations relating to education and workforce development outlined in the WHO global strategy and action plan on oral health.

## 1 Introduction

Most oral healthcare in Europe is delivered in primary care settings, namely independent general dental practice and public or community dental services (PDS or CDS) operated directly by state health authorities [1, 2]. Undergraduate education for dentists and other dental care professionals (DCPs), for example dental hygienists and therapists, has traditionally taken place within university dental teaching schools and hospitals [3]. While this model of education affords students opportunities to gain experience and competencies in a range of clinical procedures, some have suggested that the clinical environment coupled with the types of patients referred into teaching hospitals does not replicate the realities and challenges of everyday primary dental care clinical practice [4].

In its *Safe Practitioner framework* document, the General Dental Council (GDC), which regulates dental professionals in the United Kingdom (UK), outlines a series of learning outcomes and behaviours for dental professional education programmes [5]. This framework replaces the earlier *Preparing for practice* document [6] and describes the attributes expected of newly qualified dental professionals under four domains: ‘clinical knowledge and skills’, ‘interpersonal skills’, ‘professionalism’ and ‘self‐management’. The concept of ‘safe practitioner’ is multifaceted and not only includes having the requisite clinical skills and competencies to work in clinical practice, but broader behavioural, emotional, and attitudinal attributes including communication, professionalism, teamwork, leadership and management. Similarly, in its 2017 framework, *The Graduating European Dentist (GED)*, the Association for Dental Education in Europe (ADEE) emphasises the importance of developing undergraduate curricula that promote safe and effective clinical practice (including teamworking, communication, leadership and management), professionalism, patient centred care and dentistry in society [7]. *GED* highlights the need for higher education institutes (HEIs) to develop appropriate educational strategies that prepare students for independent practice and to consider different means of teaching and learning including placements in primary care settings.

Recent decades have seen the emergence of clinical education for undergraduate dental students in primary care and community (PCC) settings that are distant or separate from dental teaching hospitals but which are coordinated by traditional providers [8]. These external training placements, widely referred to as dental outreach or community‐based dental education (CBDE), were first reported in the UK in the 1970s and have since been widely adopted by HEIs internationally [9]. Outreach or CBDE teaching can take place in existing CDS or PDS clinics staffed by teams locally, in purpose built facilities designed to replicate primary dental care but staffed by dental school teams and less frequently, in general dental practices [10, 11]. During placements, students can provide episodes of supervised urgent or comprehensive care over variable time periods to patients living in the local community including adults and children [12]. Placements are generally adjunctive to core clinical training in teaching hospitals and can be block, continuous or standalone in nature with students often having dedicated nursing support [13, 14]. Other models exist including observational ‘mentoring’ models where students are linked with local practitioners but do not carry out any clinical work, hybrid models or placements in secondary care [15]. In recent decades, some HEIs have developed curricula wherein the majority of patient‐facing clinical teaching is delivered in primary care settings rather than traditional dental teaching hospitals [8, 16, 17].

The intended objectives and reported benefits of these pedagogical approaches are many [18]. In preparation for practice, they aim to foster greater personal and professional development among graduates, including increased confidence, independence, and decision making [9, 13]. It affords undergraduate students experience working in primary care and, as such, encourages an understanding and appreciation of the responsibilities and requirements of the practice environment, including team working and interprofessional working, time management, and clinical governance. Furthermore, consolidation of knowledge and development of students' clinical and communication skills are also seen as key endeavours of such placements. Finally, PCC‐based programmes place greater emphasis on developing students' ethical responsibility for the oral health of the whole community and developing their understanding of population and public dental health. During a 2004 meeting of the International Association for Dental Research (IADR), the Education Research Group considered various aspects of outreach teaching internationally, including rationale and the beneficial effects. Additional rationale for introducing such programmes included exposing students to a more diverse patient base and patients more likely to present with common health and oral health conditions [8].

Understanding how PCC‐based dental education achieves intended outcomes, and benefits students from an educational perspective, and in the broadest sense will be key in appraising the success, continued relevance, and future development of such programmes. This is of particular importance in the context of the World Health Organisation (WHO) Global oral health action plan (GOHAP) 2023–2030 [19]. This sets out a vision for the development of universal health coverage (UHC) for oral health and emphasises the need to embed oral health within primary healthcare. Notably, with respect to education and workforce, the GOHAP asks UN member states to reform oral health education to ‘prepare students for collaborative practice and integrating oral health into primary health care’ and ensure that curricula provide ‘oral health workers with clinical and public health competencies to prevent and treat the most common oral diseases with essential oral health care and rehabilitation measures in a primary care context’ [19].

Against this backdrop, the aim of this study was to identify and map reported educational outcomes associated with undergraduate dental education delivered in primary care and community‐based settings in Europe.

## 2 Material and Methods

To address our research aim, a rapid scoping review was undertaken based on Arksey and O'Malley's (2005) framework [20]. A protocol was developed a priori using the Joanna Briggs Institute (JBI) guidelines for conducting scoping reviews [21]. Scoping reviews are a form of evidence synthesis used to ascertain the nature, breadth, and depth of knowledge relevant to a broad topic or concept through searching available published literature [22]. They are useful for identifying gaps in knowledge and to inform further evidence syntheses, for example systematic reviews. Rapid scoping reviews are a variation on these, often adopted when time and resources are limited [23]. These may have a narrower scope of inquiry and limitations on searches. We examined the concept of undergraduate dental education delivered in PCC settings in the broadest sense. For this review, this included extramural placements (e.g., outreach), wholly or majority primary care‐based programmes and community engagement projects (CEPs).

Owing to the rapid nature of this scoping review, the geographical focus was limited to countries signed up to the Bologna process. This process came into effect in 1999 and established the European Higher Education Area (EHEA) to harmonise, quality assure, and ensure mutual recognition of university degree programmes across Europe [24]. The full inclusion criteria are outlined in Table 1. Ethical approval was not required for this review.

**TABLE 1:** Inclusion and exclusion criteria.

Inclusion criteria	Exclusion criteria
Peer‐reviewed journal articles Conference papers Reporting on: Undergraduate dentistry education (‘basic dental education’) Measured or recorded outcomes of dental outreach or education delivered in primary care or community settings separate from traditional teaching facilities (any type) Publicly funded universities Jurisdictions signed up to the Bologna process (EHEA members) Written in the English language Published from January 1st 2000	Opinion papers or other texts Textbook chapters Non‐peer‐reviewed articles Reporting on: Postgraduate dental education, for example, speciality training or continuing professional development (CPD) courses Privately funded universities Jurisdictions out with the Bologna process/European Higher Education Area Written in a language other than English Published before 2000

### 2.1 Search Strategy

A three‐stage search strategy was adopted as recommended in the Joanne Briggs Institute (JBI) guidelines [21]. Firstly, a limited search of two online databases, PubMed (MEDLINE) and Web of Science Core Collection, was carried out using the terms ‘dental’ and ‘outreach’. Titles and abstracts were read, and relevant terminology collated.

A second, comprehensive search of Web of Science Core Collection, Academic Search Complete, CINAHL Complete, ERIC and MEDLINE via EBSCO was undertaken in October 2022 and updated in March 2024, informed by keywords and terms identified in the initial search. The following search syntax was utilised: TOPIC: (undergraduate* OR student*) AND (dental OR dentist*) AND (outreach OR placement*). The search strategy was reviewed by a subject librarian at Trinity College Dublin.

Lastly, the reference lists of all identified articles were searched for any additional sources.

### 2.2 Study Selection and Synthesis

**FIGURE 1:**
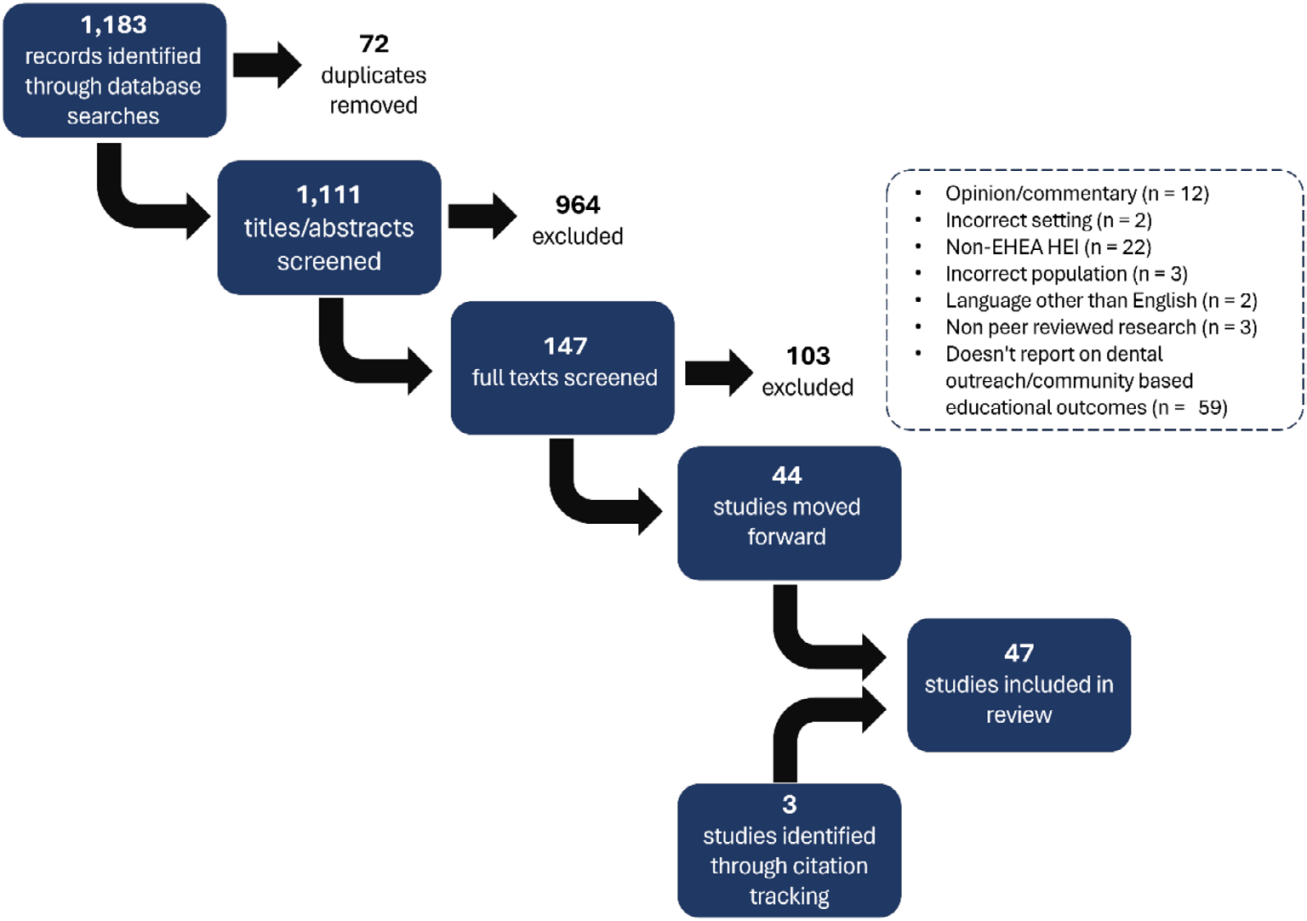
PRISMA diagram.

Search results were imported to Endnote 20 reference management software for deduplication. Thereafter, citations were uploaded to COVIDENCE online systematic review management platform for screening and selection. Titles and abstracts were firstly screened (*n* = 1111), followed by full texts of 147 potentially relevant articles (Figure 1). Data from included studies were collated using Microsoft Excel and captured the following data items: authors, year of publication, jurisdiction, and dental school(s), setting, for example, general dental practice, public dental service or purpose‐built simulation centre, educational outcomes reported, and study design including means of recording or measuring outcomes (e.g., surveys or interviews). Results were single screened by the lead author (PL) with selected studies being reviewed independently by both authors.

Critical appraisal of selected studies did not take place. In contrast to systematic reviews, this element of evidence synthesis is not considered essential for scoping reviews [22].

Educational outcomes have been synthesised narratively, grouped, and themed into five broad domains.

## 3 Results

Forty‐seven studies met the inclusion criteria and were therefore included in this review (Appendix S1). The selected papers focussed on 14 dental schools spanning the UK (*n* = 10), Germany (1), Norway (1), Finland (1) and Sweden (1). Most PCC‐based education models pertained to the final or penultimate years of study, although some studies reported on primary care‐based education during earlier years of study. Two UK universities, University of Plymouth Peninsula Dental School, and University of Central Lancashire (UCLan), and the University of Helsinki in Finland employ curricula wherein most clinical teaching takes place in PCC settings. In addition to these, a range of extramural models were reported elsewhere, including block or residential, continuous, and standalone or short placements in public or community dental services, dental access centres (DACs), purpose‐built facilities or general dental practices. Most models saw students provide care directly to patients; however, a small number were observational in nature. Studies reporting on students' participation in community engagement projects (CEPs) were also included. CEPs are focussed on social responsibility and involve students interacting with members of the local community to better understand health needs and develop appropriate health interventions [25].

Studies used a variety of measures or means of recording or evaluating educational outcomes (Appendix S1). These included randomised controlled trials (RCTs), questionnaires, interviews, written or audio‐recorded reflections, routinely collected student feedback, structured seminars, case‐based discussions, student case reports, visual analogue scales (VAS), retrospective or comparative analyses of students' clinical activity, or a combination of one or more of the above. Most studies utilised questionnaires completed by students. A summary of study characteristics is provided in Table 2.

**TABLE 2:** Summary of included studies (full list available in Appendix S1).

Study characteristics	Number of studies
*Country*	
UK	43
Germany	1
Norway	1
Finland	1
Sweden	1
*Study type*	
Qualitative	6
Quantitative	6
Mixed or multimethods	28
Randomised controlled trial (RCT)	3
Other (e.g., descriptive paper)	4
*Population of interest*	
Dentistry undergraduates only	38
Educators only (e.g., tutors, supervising dentists)	1
Recent graduates	2
Mixed population (students and educators)	6
*Year of publication*	
2000–2005	5
2006–2011	21
2012–2017	13
2018–2024	8
*Total*	47

**FIGURE 2:**
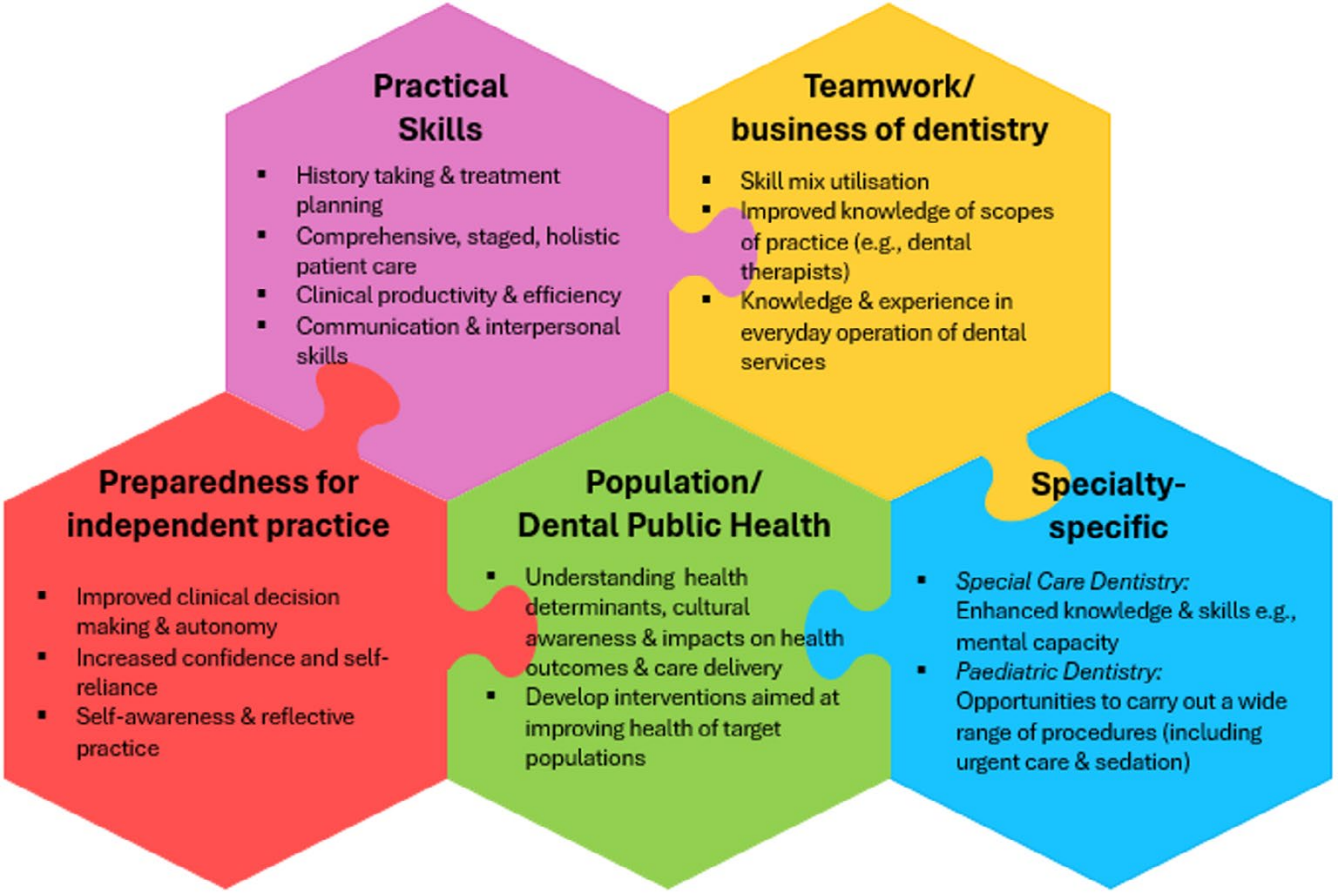
Overview of outcomes identified.

The following domains are the key findings from this scoping review (Figure 2).

### 3.1 Domain 1: Practical Skills

#### 3.1.1 Treatment Planning and Holistic Patient Care

Primary care and community‐based education was associated with exposing students to, improving their clinical and communication skills, and increasing their confidence in managing ‘real world’ patients regarded as being more representative of local communities, and patients likely to be managed by new graduates. The literature pointed to students gaining valuable experience in planning and providing comprehensive, staged and integrated patient care reflecting that delivered in general practice, often on an ongoing basis and from start to finish [9, 26, 27, 28, 29, 30, 31, 32]. PCC education was noted for enhancing undergraduates' awareness and understanding of the impact of social, economic and cultural circumstances and contexts on oral health and implications for realistic treatment planning, for example, by taking families' expectations of care and ability to attend appointments into account or the impact of dental anxiety on care delivery [33, 34, 35, 36, 37, 38, 39]. In their 2006 RCT investigating the effect of outreach on treatment planning abilities, Smith et al. found these placements were more effective in improving social history recording and translation into care planning compared to the dental hospital setting [40]. This finding was mirrored by Harris et al. [33] in their comparative analysis of students' reports focussing on the social histories of their patients within the context of the local community [33]. Outreach also led students to provide more holistic, pragmatic care for their patients, taking all their health needs into account [28, 37, 41]. This contrasts with the often secondary care‐focussed dental hospital setting where individual treatment items are often undertaken on long‐standing hospital patients to meet student treatment targets and competences [3, 18]. More broadly, PCC‐based teaching was credited with affording undergraduates' greater breadth of experience in carrying out a greater quantity and variety of routine dental procedures as well as new and alternative treatment approaches [27, 31, 34, 37, 42, 43]. Dental tutors also noted improvements in students' diagnostic, care planning, operative skills and dexterity during their placements [42].

#### 3.1.2 Clinical Productivity

Increased clinical activity and efficiency were beneficial outcomes acknowledged in the literature [27, 44]. Following a pilot placement programme in general dental practice, Craddock found the quantity of sessional clinical work carried out by Leeds University students almost trebled compared to the dental hospital setting [45]. Similarly, Smith et al. reported that block placements in primary care increased the volume of Sheffield undergraduates' clinical activity threefold, with twice the number of patients managed during outreach (including a greater proportion of children) compared to a similar period spent in hospital [31]. In the same study, students also reported gaining an appreciation for the importance of clinical priority setting. With respect to speed and efficiency, Craddock reported that 80% of Leeds students felt their time management skills had improved following placements in purpose‐built outreach facilities over a 12‐month period [38]. Similarly, almost 90% of final year students at Newcastle dental school felt primary dental care outreach (PDCO) had improved their ability to manage their clinical time effectively after 2 years [35]. Factors associated with increased productivity included students having individual nursing support, working in smaller teams, less restrictive organisational structures, and a higher throughput of patients compared to the dental school setting [31, 45].

#### 3.1.3 Communication

Several studies reported on the benefits of PCC‐based education in developing students' communication and interpersonal skills. This included with team members, patients, and the wider community [3, 11, 27, 28, 31, 37, 46, 47]. Reflecting on their time spent in primary care, final year dental students at Liverpool University reported that outreach had had a large positive impact on their interpersonal skills, including their ability to communicate effectively with a high volume of patients according to Lennon et al. [34]. Students spent one day per week for eleven weeks working between two National Health Service (NHS) dental practices in the northwest of England. Likewise, following work shadowing in general dental practices across the Frankfurt am Main area in west‐central Germany, students at the Goethe University perceived their interpersonal and communication skills had improved, in particular when interacting with patients and the practice team [15]. Using a validated instrument, students self‐assessed their core and supplementary competencies before and after their placements during which they observed experienced dentists in their everyday clinical practice. Undergraduates noted that their ability to ‘convey the content of a procedure to a patient clearly’ had improved significantly and that observing the *‘interaction of my teaching dentist with the practice team was of role model quality to me’* [15]. Outreach was also credited for honing students' ability to communicate with patients for whom English was not their first language. Craddock [38] noted that more than 95% of patients attending one outreach centre in West Yorkshire were from ethnic minorities, of whom approximately half required the assistance of an interpreter. Subsequently, this allowed students to develop a range of new communication skills, including use of translation services [38]. Community engagement was also cited for enhancing students listening skills and their awareness of nonverbal communication cues and body language [48, 49]. These skills allowed students to build trust and rapport with a diverse range of population groups including the elderly, adults with intellectual disabilities and populations experiencing homelessness [48, 49].

### 3.2 Domain 2: Teamwork and the Business of Dentistry

#### 3.2.1 Teamwork and Interprofessional Learning

A major educational outcome frequently reported in the literature was the promotion of team working skills. Students gained greater awareness of the importance of, and benefited from working with immediate dental team members and other health and social care professionals across all PCC settings [3, 9, 12, 26, 27, 28, 31, 32, 34, 35, 37, 38, 39, 42, 46, 50]. Students participating in general dental practice and public dental service‐based placements reported being swiftly and easily integrated into existing teams and perceived that they had contributed to team dynamics and the smooth operation of practices, for example by seeing emergencies and easing pressure on other clinicians [15, 34, 37, 45]. A major factor attributed to students developing their teamwork skills was working chairside with a dedicated dental nurse, a factor often absent in dental teaching hospitals [27, 34, 35, 38, 51, 52]. Working alongside a dedicated dental nurse not only afforded students experience in four handed dentistry, but students reported gaining a deeper appreciation for the important role they play [3, 12, 26, 37, 38, 39, 46].

‘I enjoyed working with the same nurse as it helped me understand the importance of having a good relationship with a nurse’ [3]

This appreciation and experience also extended to other DCPs including student dental hygienists and therapists [9, 32, 37, 42, 51, 53]. Through observation, planning and delegation of elements of care to appropriate team members, undergraduates valued other DCPs' roles in the delivery of comprehensive care to patients and enhanced their understanding of scopes of practice [46, 51].

‘*Before coming here, I had never referred patients to DHT and did not know how to. Now it's routine*’ [51]

More broadly, PCC‐based education was credited with fostering professional socialisation and collegiality among dental and other DCP students through interprofessional learning, cooperation, and shared patient care [46, 50].

#### 3.2.2 The Business of Dentistry

As highlighted above, dental undergraduates gained first‐hand experience of care delivery in different settings out with the dental hospital, including activities in nondental settings such as care homes and schools [32, 37, 42]. Students appreciated being able to compare different services and furthered their understanding of the wider context in which dentistry is practised [12, 32, 38]. Through immersive practice and experiential learning, undergraduates reported gaining knowledge, skills and experience in the everyday operation and management of primary dental care services [26, 31, 42]. Several UK studies found students gained knowledge and experience of the delivery and administration of publicly funded NHS general and community dental care, including contractual arrangements, system of remuneration and key performance indicators (KPIs) [28, 41, 54]. Delivering dental care as part of a live NHS contract also appears to have had the benefit of encouraging some undergraduates to consider a career in the public system.

‘My experience of real Units of Dental Activity and Key Performance Indicators has encouraged me to positively consider NHS high street dentistry as a career option’ [51]

Among a cohort of students who undertook part of their training in the public dental service in northern Norway, the majority took up employment in that service after qualification [42]. More generally, training in PCC settings afforded students opportunities to develop their business and clinical governance skills, for example in dental practice organisation, accounting, marketing, decontamination and reprocessing of instruments, safeguarding, and clinical audit [11, 15, 38, 46, 51, 55]. Students also gained first‐hand experience utilising different electronic patient management systems and imaging software used in primary care [9, 38].

### 3.3 Domain 3: Preparedness for Independent Practice

Primary care and community based education, in particular outreach, was noted for consolidating students' learning and helping them to understand how theory translates into everyday clinical practice [32, 34, 37, 52]. Dental students frequently reported that their time in primary and community care led to improvements in their ability to make clinical decisions and practice independently [3, 32, 35, 41, 46, 52, 54]. Undergraduates acknowledged and appreciated the support, encouragement, and respect given to them by their clinical tutors in fostering this self‐reliance and autonomy [41, 42, 46].

‘The respect from tutors when around patients really made you feel like you were in charge and making decisions’ [46]

Dental outreach was reported to have had the positive effect of promoting self‐actualisation, self‐awareness and reflective practice among undergraduates. Through greater clinical independence and development of self‐autonomy, students were better able to critically self‐appraise their own strengths and limitations and know when and how to ask for help when required [41, 46]. Perhaps one of the most cited outcomes throughout the selected studies was increased confidence [3, 9, 12, 25, 26, 27, 32, 34, 35, 37, 41, 42, 44, 46, 48, 49, 51, 52, 56, 57]. Increased confidence related to clinical ability, communication, engagement with disadvantaged groups and working with others, for example dental nurses. Once again, students cited trust and encouragement from their tutors and having dedicated chairside support as contributory factors in this regard [3, 12, 25, 41, 46, 48, 49]. In another randomised controlled trial, Smith and colleagues found that outreach placements led to a significant increase in students' clinical confidence in managing patients [56]. The study allocated a cohort of 4th year students to existing dental hospital clinics or outreach placements in primary dental care. Undergraduate supervisors also noted improved confidence and independence among students [42]. Students (and newly qualified dentists already in practice) perceived that they were better prepared for life as independent practitioners as a result of primary care placements [3, 9, 26, 28, 46]. Additional benefits were also reported, for example gaining insights into potential career pathways and getting help with preparing CVs for job applications [26, 35, 37, 39]. Furthermore, placements away from the dental school did not appear to negatively impact upon undergraduates’ final grades according to one randomised controlled trial [58].

### 3.4 Domain 4: Population and Dental Public Health

Outreach and CEPs gave undergraduates the opportunity to deepen their understanding of wider population health issues and the determinants of health [25, 33, 37]. As previously highlighted, PCC‐based education was noted for promoting students' awareness and understanding of the social, environmental, educational, economic and cultural circumstances of their patient base and the local community and the impact these had on health outcomes and care delivery. Some outreach programmes saw students undertake adjunctive project work, for example community profiles and case reports, through which they developed a greater appreciation of these issues and their social accountability [33, 36, 59]. A series of articles focusing on community engagement among students attending the Peninsula Dental School in southwest England found increased awareness among students of social inequalities and their influence on health and wellbeing [25, 47, 48]. Through direct engagement with vulnerable and socially excluded groups and collaboration with community and voluntary organisations, students gained greater insights to the challenges faced by and the complex needs of such populations [47, 48]. This also had the effect of challenging students' assumptions and sometimes negative preconceptions surrounding disadvantaged populations, for example, those experiencing homelessness [47, 48]. From a practical perspective, CEPs gave students opportunities to develop interventions aimed at improving the health of their target populations [25, 48]. This not only promoted the development of skills in dental public health but project planning and management skills [25].

### 3.5 Domain 5: Specialty Specific Outcomes

In addition to dental public health, several studies investigated and reported on outcomes of PCC‐based education relevant to the clinical specialties of special care dentistry (SCD) and paediatric dentistry [14, 60, 61, 62, 63]. These specialities are notable for being associated with public and community dental services in many European countries, where much primary dental care‐based education takes place. Following an outreach pilot in the community dental service (CDS), Sheffield University students reported improved understanding of the barriers to care faced by patients with special dental care needs and enhanced knowledge and skills in areas such as assessing mental capacity, managing patients with dementia, shared care, and referrals to specialist services [61]. Reporting on an outreach programme in SCD in Wales, Curtin found undergraduates had improved their understanding of the issues surrounding dental care delivery to groups with special dental care needs [14]. Undergraduates at Cardiff University observed patients being treated in a variety of settings including CDS clinics, a mental health facility, and in patients' own homes. Several articles reported on the benefits of dental outreach in affording students' greater opportunities to manage children and carry out a wide range of routine and more advanced paediatric dental care procedures [12, 62, 64]. These procedures included restorations on primary teeth, pulp therapies, and extractions, with students reporting an increase in confidence in undertaking specific items of care (and managing paediatric patients in general) after their placements [12, 63, 64, 65]. Outreach was credited with honing core clinical skills in paediatric dentistry, with students perceiving that they were well prepared for managing children in general dental practice on graduating [62, 64]. Some outreach programmes also allowed students to gain first‐hand experience of delivering emergency paediatric dental treatment and care under inhalation sedation [12, 63].

## 4 Discussion

### 4.1 Summary of Findings

This scoping review investigated educational outcomes associated with primary care and community‐based undergraduate dental education as reported in the European literature. Our findings highlight a range of outcomes relevant to the domains and outcomes outlined in both the ADEE's *Graduating European Dentist* and the GDC's *Safe Practitioner framework*, providing evidence for the benefits of such placements in preparing students to enter the oral health workforce and independent practice [6, 7]. We found programmes afforded students opportunities to develop their skills in communication, teamwork, time management, history taking, and treatment planning. Through engagement with local communities, programmes also afforded undergraduates opportunities to deepen their understanding of population oral health needs and explore ways to improve the health of communities through novel approaches, fostering a sense of social accountability. The literature widely reported students gaining knowledge and experience in the everyday operation of dental services, improving their productivity, decision making, and gaining in confidence. Students widely reported feeling better prepared for independent practice because of their placements.

### 4.2 Strengths and Limitations

A notable strength of this review is that it helps to fill a research gap by collating and therefore deepening our understanding of the outcomes associated with, and the potential benefits of PCC‐based education in preparing dental undergraduates for independent clinical practice. By utilising a scoping review, we were able to capture and map a broad and comprehensive range of reported outcomes across the European literature and explore ways in which undergraduate dental education can be delivered out with the ‘traditional’ dental teaching hospital model. Another strength of the review is that our searches yielded peer‐reviewed primary research studies and therefore empirical evidence, which was utilised to directly inform our research aim. As part of this review, we adhered to the Joanna Briggs Institute (JBI) scoping review guidelines to inform our methodology, including searching, reviewing and synthesising the literature.

A limitation of this, and other scoping reviews, is the absence of formal quality appraisal (QA) and methodological interrogation of studies. In the case of our review, however, all included studies were published in peer‐reviewed scientific journals. As this was a rapid scoping review, the geographical focus was limited to Europe, as was our population of interest—to dentistry students only which was to the exclusion of other dental care professional undergraduates. Furthermore, owing to time and resource constraints, a focussed search strategy of databases was adopted for this review. While a more exhaustive search strategy would have been preferable, adopting an abbreviated search strategy is one such approach that can be applied in rapid scoping reviews to identify evidence where methodological efficiencies are required [66].

Our searches revealed a paucity of literature from outside the UK, the Nordic countries and Germany. While this shortcoming may, in part, be reflective of our focussed search strategy, it is likely attributable to a lack of research in this subject area from outside these areas. These aspects may impact the generalisability of our findings to other dental undergraduate programmes, for example, in dental hygiene or therapy, and other jurisdictions. Time and resource constraints also meant that we adopted a single reviewer approach to article screening. While this is a commonly adopted approach in rapid reviews, it also introduces the possibility of screening bias and increases the risk of studies being unnecessarily excluded [66]. Another limitation of note is the heterogeneity among included studies, including in study design, types of PCC‐based programmes under investigation and outcome measures used. While some studies utilised more ‘objective’ or quantitative outcome measures, for example clinical activity or patient throughput, more frequently, outcomes were reported from the subjective perspectives of students, and without triangulation with other measures, for example competency‐based outcomes, tutors' assessments or patient reported outcomes. We found that few studies explicitly reported that they had utilised instruments or measures that had either undergone piloting or formal validation, or which were previously validated, for example, the visual analogue scale (VAS).

### 4.3 Comparison With the Existing Literature

This study builds upon and complements a series of existing reviews examining undergraduate dental education, including outreach. A 2021 scoping review examining different methods and trends in dental undergraduate teaching in the UK and Ireland listed dental outreach as one such initiative and suggested that the development of outreach centres could be regarded as the greatest innovation in clinical skills teaching in dentistry [67]. The findings of our review concur with a qualitative evidence synthesis by Ross and Holder [68], which examined undergraduate dental students' perceptions of primary care placements in the UK [68]. They found students generally valued their placements and perceived that they were better prepared for independent practice as a result. Several themes emerged from that review which contributed to the development of a conceptual model of the student experience in outreach dental education including exposure to ‘real world’ dentistry, the learning environment and support for learning, for example through having dedicated nurses and supportive tutors [68]. While that review of eleven UK‐based studies did critically and systematically appraise the literature, it focussed solely on students' perceptions about placements and was limited to UK dental schools. They found that while placements were broadly viewed positively (for reasons outlined in our review), some students highlighted competing cultures and tensions between the traditional teaching hospitals and primary or community care settings. One notable example of such tension was that of the differing treatment complexities among patients attending the different settings and the perceived difficulties in meeting clinical targets and requirements when managing cases viewed as being ‘routine’ or of low complexity [27]. Another scoping review by Elsheli et al. [69] examined the origins, intentions and motivations of CBDE programmes globally [69]. While broad in its objectives, that review uncovered and highlighted many of the same outcomes as our review in a high‐level summary [69]. Notably, many of these same outcomes were reported in the non‐European literature, for example the United States, Canada, Australia and South Africa. Other key outcomes uncovered in the non‐European literature were students' positive attitudes towards working in underserved areas and their consideration of taking up employment in remote and rural locations after qualification. These outcomes are most likely reflective of community‐based programmes being seeded in such areas, for example in Australia where rural placements feature prominently [70, 71]. Finally, in their recent scoping review examining students' and supervisors' perceptions of CBDE, Taylor et al. [72] not only identified a myriad of positive outcomes mirroring those of our review, they also highlighted some negative perceptions among students and supervisors alike [72]. Among students, this included frustration with inconsistencies in teaching and assessment between dental school and outreach settings.

### 4.4 Policy and Practice Implications

This scoping review is timely as health systems look to develop and widen access to universal, affordable primary oral healthcare, reduce oral health inequities and meet international obligations commensurate with the WHO GOHAP [19]. Embedded within the action plan is the integration of oral health into primary care and developing and empowering the workforce to deliver essential packages of care for all population groups, including the vulnerable and marginalised. To meet these obligations, member states are asked to reform dental education to prioritise competencies in public health, health promotion, disease prevention and the social and commercial determinants of health. Additionally, the action plan asks member states to reform education so that graduates have the competencies to manage the most common oral diseases in the context of primary care. These aspirations are particularly apt at the current time when health systems are experiencing workforce shortages in oral healthcare, notably in state funded services, for example, NHS primary dental care services in England [73]. The findings of this scoping review demonstrate how PCC‐based undergraduate dental education can help member states and education providers achieve these goals. By seeding clinical teaching in these settings, including in areas of high unmet needs, students are afforded greater exposure to higher volumes of disease and first‐hand experience of routine care delivery in settings which are the same as or similar to those in which many of them will practise after graduation. For many students, undertaking such placements not only has the potential to hone their clinical and professional skills, but develop positive attitudes towards primary care dentistry and the provision of state‐funded care. From a workforce planning and oral health policy perspective, this has the potential to leverage new graduates' engagement in these services. Maximising the appeal of routine and state‐funded primary dental care is arguably more important than ever as the oral healthcare landscape experiences greater diversification where the demand for, and commodification of highly remunerative ‘non routine’ care including facial aesthetics and cosmetic dentistry is growing [74, 75] and new graduates, who increasingly value career variety and part‐time practice [76], face substantial debts to repay amidst increasing professional and living costs [73, 77].

The outcomes of this review are consistent with and appear to meet the objectives and intended learning outcomes of many PCC‐based programmes, for example those outlined in the ADEE Special Interest Group (SIG) on outreach teaching 2011 report [13]. While these placements bring additional benefits to undergraduate programmes, HEIs must consider additional programme costs, for example, commissioning and constructing new teaching facilities, hiring and paying clinical and administrative staff as well as procuring equipment and materials. Additionally, when planning where to locate outreach or other PCC‐based education facilities, there is a need to ensure that these are in areas where the required or anticipated throughput of suitable patients can be guaranteed, for example, in underserved communities or areas of high unmet need and which are readily accessible, for example, by public transportation. As highlighted in this review, PCC‐based education can be beneficial in fostering collaborative practice and interprofessional working, including skill mix utilisation and role delegation among dental and DCP students. This is important in the context of the WHO GOHAP's call for the development of innovative workforce models, where collaborative practice both within and out with dental teams is promoted [19]. Where opportunities for the development of interprofessional learning and working in PCC‐based programmes arise, these could and should be explored.

Finally, consideration must be given to quality assurance (QA) and clinical governance challenges, which can arise from the requirement to balance educational objectives with the delivery of high‐quality patient care across different clinical settings. Challenges can include ensuring there is sufficient patient volume, case mix, adequate training of clinical tutors to maintain uniformity in standards of supervision, teaching, and assessment, ensuring consistency in protocols and procedures across various sites, for example, infection control, as well as equipment and materials to meet educational needs [78]. Where PCC‐based teaching coexists alongside the established dental teaching hospital model, programme planners and educators must recognise and address any competing tensions through ongoing curriculum review and design that incorporates and blends the various elements effectively and through ongoing staff development. As highlighted in this review, ensuring there is consistency in educational quality across the different settings and assessing whether the objectives of such programmes are being met can be somewhat challenging. HEIs therefore need to put in place robust systems of audit, feedback, and assessment [78].

## 5 Conclusion

Primary care and community‐based undergraduate dental education including outreach facilitates dental students in consolidating their learning and honing their skills in preparation for independent clinical practice. Educational outcomes cited in the European literature align with many of the objectives and intended learning outcomes of such programmes, as well as the ADEE's *Graduating European Dentist* curriculum framework. To deliver on the WHO global strategy and action plan on oral health, namely the development of universal health coverage for oral health, embedding oral health within primary care and strengthening systems to better serve population needs, UN member states will need to enhance the capacity and capabilities of their respective oral health workforce. To do this, HEIs, with the support of policymakers and wider government, will need to review and develop their undergraduate curricula and programmes to ensure that they are fit for purpose in preparing new graduates for the realities of contemporary primary care practice. This review highlights the importance and benefits of primary care and community‐based undergraduate dental education in supporting these endeavours.

## Conflicts of Interest

The authors declare no conflicts of interest.

## Supporting information


Appendix S1.

## Data Availability

The data that supports the findings of this study are available in the supplementary material of this article.
