# Supplementary material for: Educational Outcomes Associated With Undergraduate Dental Education in Primary Care and Community Settings in Europe: Findings From a Rapid Scoping Review
Source: Eur J Dent Educ. 2025 Jun 30;30(2):578–89. doi: 10.1111/eje.70004 (PMC13090436; doi:10.1111/eje.70004)
Supplement: Supplementary file 1 — Appendix S1. [file EJE-30-578-s001.docx]

**Supplemental data file**

**Table 1: Characteristics of included studies (in order of citation)**

| **Study title** | **Authors, Year** | **Country, HEI** | **Primary care/community-based education model** | **Population and number (n)** | **Instruments/outcome measures used** |
| --- | --- | --- | --- | --- | --- |
| Student perspectives and opinions on their experience at an undergraduate outreach dental teaching centre at Cardiff: a 5-year study | Lynch et al., 2010a | UK, University of Cardiff | Outreach: purpose-built unit | Dental students (n=257) | Survey: primarily quantitative with some open-ended questions |
| Outreach clinical dental education: the Portsmouth experience - a 4-year follow-up study | Radford et al., 2016 | UK, Kings College London | Outreach – purpose built (University of Portsmouth Dental Academy), block residential | Dental students (n=303) | Student questionnaire (quantitative and qualitative) |
| A dental practice placement scheme: benefits for practitioners and undergraduates | Pau and Croucher, 2001 | UK, St Bartholomew’s and The Royal London School of Medicine and Dentistry | General Dental Practice, observational placement | Dental students (n=61)  GDPs (n=34) | GDP and student questionnaires and structured seminars |
| Community Health Centre-Based Outreach Clinic for undergraduate dental education: Experience in Helsinki over 8 years | Goswami et al., 2018 | Finland, University of Helsinki | Outreach – Community Health Centre | Dental students, n=150 | Annual online survey (quantitative and qualitative) |
| Evaluation of an observational outreach programme in Special Care Dentistry for undergraduate students; reflections over three years | Curtin and Thompson, 2015 | UK, University of Cardiff | Observational outreach in primary care settings – CDS | Dental students, n=67 | Analysis of students’ reflective logbooks (quantitative and qualitative) |
| Work shadowing in dental teaching practices: evaluation results of a collaborative study between university and general dental practices | Heitcamp et al., 2018 | Germany, Goethe-University Frankfurt-on-Main | General Dental Practice, observational placement | Dental students, n=61 | Student questionnaires and written reports (students and supervising dentist) |
| The benefits of an innovative community engagement model in dental undergraduate education | Witton and Paisi, 2022 | UK, University of Plymouth – Peninsula Dental School | Community Engagement Project (CEP) | Students (70-80 per year) | Descriptive analysis of routinely collected student feedback |
| Perceptions of a simulated general dental practice facility - reported experiences from past students at the Maurice Wohl General Dental Practice Centre 2001-2008 | Davies et al., 2009 | UK, Kings College London | Outreach – General Dental Practice | New graduates, 6 months post qualification, n=136 | Questionnaire: quantitative and qualitative, Visual Analogue Scale (VAS) |
| Developing dental education in primary care: The student perspective | Elkind et al. 2005a | UK, University of Manchester | Outreach – CDS and personal dental services (PDS) pilot site | Dental students, n=48 | Questionnaire: open questions |
| In real life: evaluating primary care-based undergraduate dental education in Northwest England | Kuroski et al., 2019 | UK, University of Central Lancashire (UCLan) | Dental Education Centres | Dental students, n=5 | Semi-structured interviews |
| Developing the continuum of dental education: including dental foundation trainers in the delivery of a community-based clinical teaching programme | Lynch et al., 2012 | UK, University of Cardiff | Outreach: purpose-built unit | Dental Foundation (DF) trainers, n=20 | Questionnaire: open questions |
| Patients treated by dental students in outreach: the first year of a pilot project | Elkind et al., 2005b | UK. University of Manchester | Outreach – CDS and personal dental services (PDS) pilot site | Dental students, n=not specified | Summary descriptive statistics drawn from treatment summaries and questionnaires. |
| Students’ clinical experience on outreach placements | Smith et al., 2010 | UK, University of Sheffield | Outreach – CDS, DAC or General Dental Practice (and dental hospital- for comparative purposes) | Dental Students, n=173 | Quantitative assessment of clinical records and questionnaire (Likert scales) |
| Evaluation of an outreach education model over five years: Perception of dental students and their outreach clinical mentors | Leisnert et al., 2017 | Sweden, Malmö University | Outreach: Swedish Public Dental Service (PDS), continuous | Dental students, n=85  Tutors, n=74 | Questionnaires (Likert scales and free texts) |
| Recording and understanding social histories by dental undergraduates in a community-based clinical programme | Harris et al., 2003 | UK, University of Liverpool | Outreach – CDS, continuous | Dental students, n=103 | Student written report on profile of local community and case histories of two patients. Assessed by two calibrated assessors. |
| The personal dental service as a setting for an undergraduate clinical programme | Lennon et al., 2004 | UK, University of Liverpool | Outreach: General Dental Practice (Personal Dental Service), continuous for 11 weeks | Dental students, n=6 | Questionnaires: Likert scales, interviews and analysis of students’ clinical records |
| Developing a primary dental care outreach (PDCO) course – part 2: perceptions of dental students | Maguire et al., 2009 | UK, University of Newcastle | Outreach - purpose built | Dental students, n = not specified | Questionnaires: Likert scales and free texts (used routinely for student assessment) |
| Developing an assessment in dental public health for clinical undergraduates attending a primary dental care outreach programme | Holmes et al. 2011 | UK, University of Newcastle | Outreach - Salaried Dental Service | Dental students, n=71 (written report), n=60 (questionnaire)  Clinical teaching/academic staff, n= not specifed | Student written report and questionnaire. Teaching staff took part in focus group discussion (FGD) |
| Student perspectives on their recent dental outreach placement experiences | Smith et al., 2006a | UK, University of Sheffield | Outreach – CDS and DACs, block | Dental students, n=10 | Semi structured interviews and FGDs, triangulation with clinical records |
| Outreach teaching - the Leeds experience: reflections after one year | Craddock 2008 | UK, University of Leeds | Outreach - purpose-built | Dental students, n=48 | Questionnaires: Likert scales |
| 'The dental nurse played a vital role in helping to manage the patient': a thematic analysis of undergraduate dental students' reflective journals from outreach placements | Dyer and Chapple, 2023 | UK, University of Sheffield | Outreach | Dental students, n = 51 | Documentary and thematic analysis of dental students’ reflective journals |
| A randomised controlled trial of the effect of outreach placement on treatment planning by dental students | Smith et al., 2006b | UK, University of Sheffield | Outreach – CDS and DACs, block | Dental students, n=49 | Randomised Controlled Trial (RCT) |
| Empowerment in a model of outreach undergraduate dental education | Radford and Hellyer 2017 | UK, Kings College London | Outreach – purpose built (University of Portsmouth Dental Academy), block residential | Dental students, n=143 | Questionnaire: quantitative and qualitative |
| Evaluation of a dental outreach teaching programme | Eriksen et al., 2011 | Norway, University of Tromsø | Outreach – purposes built | Dental students, n = not specified | Written reflections, signed by tutors and interviews: students and tutors |
| Developing a primary dental care outreach (PDCO) course –part 1: practical issues and evaluation of clinical activity | Hind et al., 2009 | UK, University of Newcastle | Outreach - purpose built | Dental students, n=not specified | Descriptive analysis of students’ daily clinical activity (items of treatment carried out) in outreach and dental hospital |
| In their own words: investigating the preparedness of final year dental students in the UK for independent general dental practice | Ray et al., 2018 | UK, three unspecified HEIs | Outreach – CDS,  Dental Education Centres,  General Dental Practice placements | Dental students, n=17  UELs (undergraduate educational leads), n=3 | Focus group discussions and semi-structured interviews |
| An evaluation of student, patient and practitioner experience of general dental practice placements | Craddock 2011 | UK, University of Leeds | Outreach – General Dental Practice, continuous (fortnightly) | Dental students, n=19  General Dental Practitioners, n=15 | General Dental Practitioner and student questionnaires |
| Belongingness in undergraduate dental education | Radford and Hellyer 2016 | UK, Kings College London | Outreach – purpose built (University of Portsmouth Dental Academy), block residential | Dental students, n=76 | Questionnaire: quantitative (Likert scales) and qualitative (free texts) |
| ‘Today’s shook me up a lot inside…it’s definitely changed me’: emotional responses and transformative learning through working with disadvantaged communities | Neve et al. 2020 | UK, University of Plymouth – Peninsula Dental School | Community Engagement Project (CEP) | Dental students, n=8 | Audio diary reflections |
| A dental student view on learning gained through Inter-Professional Engagement with people experiencing homelessness | Webb et al. 2019 | UK, University of Plymouth – Peninsula Dental School | Community Engagement Project (CEP) | Dental students, n = not specified | Published report of dental student experience |
| Reflections on learning and enhancing communication skills through community engagement: a student perspective | Hanks et al., 2016 | UK, University of Plymouth – Peninsula Dental School | Community Engagement Project | Dental students, n=not specified | Student reflections, posters, written reports and summative assessments |
| Attitudes of the first cohort of student groups trained together at the University of Portsmouth Dental Academy towards dental interprofessional education | Colonio Salazar et al. 2017 | UK, Kings College London | Outreach – purpose built (University of Portsmouth Dental Academy), block residential | Dental students, n=52 | Questionnaire: validated 19-item instrument |
| The impact of integrated team care taught using a live NHS contract on the educational experience of final year dental students | Radford et al. 2014 | UK, Kings College London | Outreach – purpose built (University of Portsmouth Dental Academy), block residential | Dental students, n=227 | Questionnaire: quantitative and qualitative (Likert scales and free texts) |
| Evaluation of a U.K. Community-Based Clinical Teaching/Outreach Program by Former Dental Students Two and Five Years After Graduation | Lynch et al. 2010b | UK, University of Cardiff | Outreach- purpose built, continuous | Recent graduates, n=58 | Questionnaire: open and closed questions |
| Dental skill mix: a cross-sectional analysis of delegation practices between dental and dental hygiene-therapy students involved in team training in the South of England | Wanyoni et al. 2014 | UK, Kings College London | Outreach – purpose built (University of Portsmouth Dental Academy), block residential | Dental students, n=not specified | Retrospective analysis of patient records (secondary data analysis) |
| Dental students' perceptions of their experience at a residential outreach centre | Radford and Hellyer, 2015 | UK, Kings College London | Outreach – purpose built (University of Portsmouth Dental Academy), block residential | Dental students, n=66  Tutors, n=8 | Questionnaires (developed by Delphi consensus) |
| Preparing dental students for careers as independent dental professionals: clinical audit and community-based clinical teaching | Lynch et al. 2011 | UK, University of Cardiff | Outreach – purpose built, continuous | Dental students, n=n/a | Overview of audit outcomes |
| A Randomized Controlled Trial of Outreach Placement's Effect on Dental Students’ Clinical Confidence | Smith et al. 2006c | UK, University of Sheffield | Outreach – CDS and DACs, block | Dental students, n=49 | Randomised Controlled Trial (RCT) |
| Effect of Community-Based Clinical Teaching Programs on Student Confidence: A View from the United Kingdom | Lynch et al. 2010c | UK, University of Cardiff | Outreach- purpose built, continuous | Dental students, n=47 | Questionnaire: quantitative- 5-point Likert scale |
| RCT of the effects of block absence for outreach placements on dental students’ finals grades. | Smith et al., 2009 | UK, University of Sheffield | Outreach – CDS and DACs, block | Dental students, n=49 | Randomised Controlled Trial (RCT) |
| Teaching dental public health to undergraduates using community profiles and patient case studies | Nandakumar and Robinson 2011 | UK, University of Sheffield | Outreach | Dental students, n=1 | Educational case study |
| Student evaluation of clinical outreach teaching in Community Special Care Dentistry | Zoitopoulos et al. 2007 | UK, Kings College London | Outreach- CDS (special care dentistry service), one-day clinic visit | Dental students, n=120 | Student evaluation reports (quantitative and qualitative data), student essays and student feedback (content analysis) |
| Dental student evaluation of a special care dentistry outreach pilot | Bustin and Temple 2017 | UK, University of Sheffield | Outreach - CDS | Dental students, n=11 | Case-based discussions and questionnaires (Likert scales) |
| Undergraduate experience and self-assessed confidence in paediatric dentistry: comparison of three UK dental schools | Rodd et al. 2010 | UK, Liverpool, Manchester and Sheffield | Outreach:  Liverpool: primary care settings, continuous  Manchester: not specified, continuous  Sheffield: not specified, block | Dental students, n=147 | Questionnaire and VAS |
| Paediatric dentistry in outreach settings: an essential part of undergraduate curricula? | Hunter and Chaudry 2009 | UK, University of Cardiff | Outreach – CDS | Dental students, n=55 | Retrospective/secondary analysis of student treatment logbooks |
| Undergraduates' self-reported clinical experience, confidence and perspectives of hospital and outreach paediatric dentistry: a three-year multi-centre evaluation | Walley et al. 2014 | UK, Universities of Liverpool, Manchester and Sheffield | Outreach | Dental students, n=525 | Questionnaire, VAS and written reflections |
| The effect of a community dental service outreach programme on the confidence of undergraduate students to treat children: a pilot study | Hunter et al. 2007 | UK, University of Cardiff | Outreach - CDS | Dental Students, n=18 | Questionnaires: Likert scales |

CDS: Community Dental Service; PDS: Public Dental Service; NHS: National Health Service; GDP: General Dental Practitioner; VAS: Visual Analogue Scale; DAC: Dental Access Centre; UEL: undergraduate educational lead; RCT: Randomised Controlled Trial; CEP: Community Engagement Project; FGD: focus group discussion; PDCO: Primary Dental Care Outreach
